# HPV-Driven Cervical Carcinogenesis: Genetic and Epigenetic Mechanisms and Diagnostic Approaches

**DOI:** 10.3390/ijms27020803

**Published:** 2026-01-13

**Authors:** Evangelia Legaki, Theofania Lappa, Konstantina-Lida Prasoula, Zoi Kardasi, Emmanouil Kalampokas, Theodoros Kalampokas, Maria G. Roubelakis, Ekaterina Charvalos, Maria Gazouli

**Affiliations:** 1 Laboratory of Biology, Department of Basic Biological Science, School of Medicine, National and Kapodistrian University of Athens, 11527 Athens, Greece; lia.lgk89@gmail.com (E.L.); thenia412@gmail.com (T.L.); pralid18@gmail.com (K.-L.P.); zkardasi@med.uoa.gr (Z.K.); roubel@med.uoa.gr (M.G.R.); 2 Unit of Gynecologic Oncology, Second Department of Obstetrics and Gynecology, Aretaieion Hospital, 11528 Athens, Greece; m.kalampokas@gmail.com; 3 Unit of Obstetrics and Gynecology, Second Department of Obstetrics and Gynecology, Aretaieion Hospital, 11528 Athens, Greece; kalamp@yahoo.com; 4 Department of Molecular Diagnosis, Reprogenetica, Dios 44, 15235 Vrilissia, Greece; ehmc@ehmc.lt

**Keywords:** cervical cancer, HPV, genetics, epigenetics, DNA methylation

## Abstract

Cervical cancer remains a major global public health concern, with persistent infection by high-risk human papillomavirus (hrHPV) types recognized as the primary etiological factor. This review explores the multifactorial nature of the disease, focusing on the complex interplay between host genetic susceptibility and epigenetic alterations that drive cervical carcinogenesis. Evidence from genome-wide association studies (GWAS) is discussed, highlighting the contribution of specific genetic loci, predominantly within the HLA region, to susceptibility to HPV infection and disease progression. In parallel, the review examines the molecular mechanisms by which the viral oncoproteins E6 and E7 promote genetic instability and epigenetic reprogramming, including histone modifications and dysregulation of non-coding RNAs. Particular emphasis is placed on DNA methylation, affecting both the viral genome and host genes such as FAM19A4, CADM1, PAX1, and MAL, as a promising biomarker for triage and detection of high-grade intraepithelial lesions (CIN2+). Finally, the review evaluates currently available methylation-based assays and self-sampling devices, highlighting their potential to enhance diagnostic accuracy and increase participation in cervical cancer screening programs.

## 1. Introduction

Cervical cancer (CC) remains a major public health challenge, representing the fourth most common malignancy and the third leading cause of cancer-related mortality among women worldwide. In 2022, its global incidence exceeded half a million cases, ranking ninth in overall female cancer deaths [1]. Despite being largely preventable through vaccination and screening, cervical cancer continues to impose a disproportionate burden in low- and middle-income countries, where approximately 85% of new cases and 94% of related deaths occur annually. The disease accounts for an estimated 662,044 cases and 348,709 deaths [2]. Persistent infection with high-risk human papillomavirus (hrHPV) types, particularly HPV-16 and HPV-18, is recognized as the primary etiologic factor in the pathogenesis of cervical cancer [3].

More than 90% of HPV infections resolve spontaneously within 6–18 months; however, persistent infection is a necessary prerequisite for progression to cervical intraepithelial neoplasia (CIN) and, ultimately, invasive carcinoma [4]. CIN1 generally reflects transient infection, CIN2 represents lesions with variable malignant potential, and CIN3 constitutes the most clinically significant precursor to invasive disease [5].

Histologically, cervical cancer is predominantly squamous cell carcinoma, accounting for approximately 80–85% of cases, while adenocarcinoma comprises around 5% of invasive cervical cancers globally, with a rising incidence in certain populations. Both histological subtypes arise from precursor lesions, including cervical intraepithelial neoplasia and carcinoma in situ (CIS). Prognosis is largely determined by disease stage, patient comorbidities, and access to timely, evidence-based care. Five-year relative survival rates reach approximately 92% for early-stage disease but decline to 60% with regional spread and to 19% in metastatic cases [6].

Although the vast majority of cervical squamous cell carcinomas are associated with hrHPV infection, a small subset of tumors appears to be HPV-independent. These cases are more frequently non-squamous histological subtypes, such as gastric-type adenocarcinoma, clear cell carcinoma, and mesonephric carcinoma, and exhibit distinct molecular characteristics compared with HPV-associated tumors [7]. Mutations in oncogenes including TP53, PIK3CA, STK11, and KRAS have been implicated in their pathogenesis [8,9]. Importantly, some reported HPV-independent cases may reflect diagnostic misclassification, as endometrial or metastatic adenocarcinomas can mimic primary cervical lesions, underscoring the need for accurate histopathological assessment [7].

From an epidemiological perspective, the global prevalence of hrHPV infection is estimated at approximately 10.4%, reaching up to 36.5% in developing regions [10]. Established risk factors include early onset of sexual activity, multiple sexual partners, immunosuppression (e.g., HIV infection), exposure to diethylstilbestrol (DES), and a history of high-grade cervical lesions such as CIN2 or CIN3 [11]. Age is also an important determinant, with HPV infection rates peaking among young, sexually active women aged 18–30 years and declining thereafter, whereas invasive disease typically presents later in life due to the prolonged natural history of persistent infection [10].

Screening and vaccination have markedly improved cervical cancer prevention. Contemporary guidelines recommend high-risk HPV testing as the preferred screening strategy. In the United States, the 2024 USPSTF draft proposes cytology alone every three years for women aged 21–29, and primary hrHPV testing every five years for those aged 30–65, with co-testing or cytology alone as acceptable alternatives [12]. The 2025 ASCCP guidelines incorporate self-collected vaginal samples for hrHPV testing, expanding screening accessibility [13,14].

At the global level, the World Health Organization advocates HPV DNA testing as the preferred screening approach, with shorter screening intervals recommended for women living with HIV. Novel triage strategies, including DNA methylation–based assays, are currently under evaluation to improve specificity following a positive HPV test. [15]

Although the causal role of HPV in cervical carcinogenesis is well established, not all infections lead to malignancy. Persistent HPV infection induces cellular and molecular alterations that can disrupt genomic stability and promote neoplastic transformation. These processes involve both genetic mutations and epigenetic reprogramming, which together underpin the transition from infection to cancer [16]. The following sections of this review explore the genetic background of cervical cancer development and the epigenetic mechanisms that drive HPV-mediated oncogenesis.

## 2. Heredity and Genetic Factors in Cervical Cancer

The contribution of genetic factors to the development of high-grade cervical intraepithelial neoplasia (HSIL) and invasive cervical cancer remains incompletely understood. Nonetheless, evidence of familial clustering suggests that inherited susceptibility may play a role in disease risk. Families with multiple affected individuals are rarely reported, indicating that highly penetrant mutations are uncommon and that most hereditary effects are likely mediated by common variants of low to moderate penetrance that interact with persistent HPV infection. While HPV infection is the decisive etiological factor, advances in genomic technologies have enabled a more refined investigation of host genetic susceptibility. Genetic analyses estimate that inherited factors may account for approximately 27–36% of the variability in cervical cancer risk, largely attributable to common autosomal single-nucleotide polymorphisms (SNPs) with low penetrance [17]. In addition, pan-cancer heritability estimates derived from the UK Biobank and GERA cohorts suggest an overall heritability of approximately 7% for cervical cancer, comparable to that observed for ovarian and colorectal cancers [18].

Studies based on candidate genes have focused primarily on genes related to the cell cycle, DNA repair, and immune response. These include TP53, MDM2, ATM, BRIP1, CDKN1A, CDKN2A, FANCA, XRCC1, XRCC3, as well as genes of immune significance such as CD83, CTLA4, TNFA, interleukins, TGFB1, and IFNG. The development of high-throughput technologies has enabled the detection of new risk variants, paving the way for a more complete understanding of genetic predisposition to cervical cancer [19].

## 3. GWAS

Over the past decade, genome-wide association studies (GWAS) have consistently demonstrated that genetic susceptibility to cervical cancer is dominated by immune-related loci, particularly within the major histocompatibility complex (MHC) on chromosome 6p21. The most robust and reproducible associations map to the HLA region, underscoring the central role of antigen presentation and immune recognition in HPV persistence and disease progression [19,20].

Across diverse populations, variants within the 6p21.32–6p21.33 region show the strongest effects, with repeated associations involving HLA class I and II genes, including HLA-DQA1, HLA-DQB1, HLA-DRB1, HLA-B, as well as stress-induced ligands such as MICA and MICB. Fine-mapping analyses further indicate that specific amino acid residues within HLA molecules contribute to differential immune responses to HPV infection, reinforcing the functional relevance of these loci in cervical carcinogenesis [19].

Beyond the HLA region, GWAS have identified additional non-MHC susceptibility loci, although with more modest effect sizes and lower consistency across ethnic groups. These loci implicate genes involved in epithelial differentiation, inflammation, apoptosis, and cell-cycle regulation, including *PAX8/PAX8-AS1*, *CLPTM1L*, *GSDMB*, *EXOC1*, and *ADGRV1*. Population-specific studies have expanded this landscape, with loci such as *EXOC1* and *GSDMB* reported in East Asian cohorts, while large-scale analyses in European and multi-ethnic populations have confirmed both known immune-related regions and novel signals [18,20,21,22,23,24].

Cross-trait and pan-cancer GWAS further suggest partial overlap between cervical cancer susceptibility and other malignancies, identifying pleiotropic loci such as *INS–IGF2*, *SOX9*-proximal regions, *GABBR2*, *SH3GL3/BNC1*, and *TET2*. These findings indicate shared biological pathways related to cellular proliferation, differentiation, and genomic stability, extending cervical cancer risk beyond HPV-specific mechanisms [18,25].

Overall, GWAS findings support a polygenic model of cervical cancer susceptibility in which immune-related loci—particularly within the HLA region—play a dominant role, while non-HLA variants act as modifiers of risk. Rare high-penetrance mutations, such as those affecting PTPN14, likely contribute to disease predisposition in a limited subset of families or populations. Integrating GWAS signals with functional approaches, including eQTL analyses, chromatin profiling, and CRISPR-based validation, will be essential to distinguish causal variants from statistical associations and to clarify how host genetic architecture interacts with persistent HPV infection to drive cervical carcinogenesis.

While GWAS have primarily identified genetic variants that influence host susceptibility to HPV persistence and cervical cancer risk, these variants rarely act in isolation. Many risk loci, particularly within immune-related genes and regulatory regions, are thought to exert their effects by modulating gene expression rather than protein structure. This regulatory influence frequently operates through epigenetic mechanisms, including DNA methylation, histone modifications, and non-coding RNA regulation. In the context of persistent HPV infection, host genetic variation may therefore shape the epigenetic landscape of cervical epithelial cells, predisposing them to virus-driven epigenetic reprogramming and malignant transformation. This genetic–epigenetic interplay provides a mechanistic framework linking inherited susceptibility with the epigenetic alterations that characterize cervical carcinogenesis.

## 4. Mechanisms of HPV-Driven Genetic and Epigenetic Alterations in Cervical Carcinogenesis

Human papillomavirus (HPV)–driven cervical carcinogenesis arises from a complex interplay of genetic disruption and epigenetic reprogramming initiated by persistent infection with high-risk HPV types, predominantly HPV16 and HPV18. The viral oncoproteins E6 and E7 (Table 1) play a central role in this process by inducing profound alterations in host genomic stability and chromatin architecture [26,27]. Following infection, HPV exerts potent genetic effects on cervical epithelial cells by targeting key regulators of cell proliferation, apoptosis, and DNA repair. Specifically, E6 promotes ubiquitin-mediated degradation of the tumor suppressor p53, thereby inhibiting apoptosis and allowing the survival of cells harboring DNA damage, while E7 inactivates the retinoblastoma protein pRb, releasing E2F transcription factors (Table 1) that drive the expression of genes required for S-phase entry and uncontrolled cell cycle progression [26,27]. The coordinated inactivation of these tumor suppressive pathways promotes genomic instability, increasing the likelihood of replication errors and the accumulation of oncogenic alterations. With persistent infection, HPV DNA frequently integrates into the host genome, often disrupting the E2 gene, a key negative regulator of E6 and E7 expression. Loss of E2 function leads to sustained oncoprotein overexpression and further genomic destabilization, thereby accelerating progression from low-grade lesions to high-grade cervical intraepithelial neoplasia and ultimately invasive carcinoma [28,29]. In addition, host genetic variation contributes to interindividual differences in disease progression, as polymorphisms in genes involved in oxidative stress responses, immune surveillance, and DNA repair modulate susceptibility to persistent HPV infection and malignant transformation [30].

Alongside these genetic disruptions, epigenetic alterations play a decisive role in HPV-driven carcinogenesis. This is compounded by chromosomal aberrations such as amplifications, deletions, and translocations that emerge in response to viral integration, further undermining genome integrity and enhancing malignant potential [29].

DNA methylation represents one of the earliest and most clinically informative epigenetic alterations in HPV-driven carcinogenesis and is discussed in detail in the following section. Beyond DNA methylation, HPV oncoproteins extensively remodel histone post-translational modifications (PTMs)—including acetylation, methylation, phosphorylation, and ubiquitination—through direct and indirect interactions with chromatin-modifying enzymes [31,32,33,34]. Histone acetylation is particularly affected, as E6 interferes with p53 acetylation by binding to CBP/p300 [35,36], while E7 recruits histone deacetylases through the NuRD complex, leading to deregulation of E2F2 activity and enhanced HIF-1α–driven transcriptional responses under hypoxic conditions [37,38,39,40]. HPV oncoproteins also disrupt histone methylation patterns by altering Polycomb group protein function, resulting in the loss of repressive H3K27me3 marks at developmental loci, such as HOX genes, and their aberrant deposition at cell cycle–related genes. In parallel, dysregulation of activating marks such as H3K4me3 promotes angiogenesis through upregulation of HIF1A and VEGF signaling pathways [41,42,43,44,45,46,47]. These epigenetic changes intersect with abnormal histone phosphorylation signals, including persistent γ-H2AX formation induced by E7-mediated impairment of homologous recombination, which contributes to genomic instability and radioresistance [47,48,49,50,51].

Histone ubiquitination is also significantly affected in HPV-associated malignancies. HPV E6 exploits the E6AP/USP46 complex to promote degradation of key regulators such as p53, TIP60, and Set8, while E7 disrupts pRb function and RNF168-mediated DNA damage repair, alterations that are associated with adverse clinical outcomes [29,52,53,54,55,56,57].

Non-coding RNAs represent an additional critical regulatory layer in HPV-driven oncogenesis. The E6 and E7 oncoproteins modulate oncogenic microRNAs, such as miR-21, and suppress tumor-inhibitory miRNAs, including miR-34a, primarily through p53 degradation [58,59,60,61]. Moreover, long non-coding RNAs such as HOTAIR, NEAT1, and SNHG12—often upregulated through E6/E7 and c-Myc signaling—promote malignant progression by interacting with PRC2 or activating oncogenic WNT and PI3K pathways [62,63,64]. Circular RNAs further contribute to tumor biology; for example, HPV16 circE7 impairs antitumor immunity by reducing H3K27ac levels and weakening CD8⁺ T-cell responses, whereas oncogenic circRNAs such as circ_0067934, circCLK3, and circAGFG1 activate YAP, ERK, and FoxM1 signaling. In contrast, tumor-suppressive circRNAs, including circ-NOLC1 and hsa_circ_0043280, inhibit proliferation and metastasis [65,66,67,68,69].

These epigenetic alterations do not operate in isolation but instead converge to reinforce stable oncogenic transcriptional programs. DNA methylation, mediated by DNMT1 and DNMT3A/B and incompletely reversed due to impaired TET activity, acts synergistically with repressive histone modifications to silence tumor suppressor genes and regulatory microRNAs, establishing a self-reinforcing epigenetic circuitry characteristic of cervical cancer [16,46,70,71,72,73,74]. Collectively, these multilayered genetic and epigenetic alterations—driven and amplified by persistent HPV infection—form a highly coordinated oncogenic program that governs disease initiation, progression, and therapeutic resistance, while simultaneously providing a mechanistic foundation for the development of improved diagnostic biomarkers, prognostic tools, and targeted epigenetic therapies aimed at intercepting HPV-mediated malignant transformation.

**Table 1:** Key Viral and Host Genes Implicated in HPV-Induced Cervical Carcinogenesis.

Gene/Viral Protein	Type	Main Function/Role in Carcinogenesis	Reference
E6	Viral oncoprotein	Promotes degradation of p53, inhibiting apoptosis and allowing survival of DNA-damaged cells.	[26,27]
E7	Viral oncoprotein	Binds and inactivates pRb, releasing E2F transcription factors that drive uncontrolled cell cycle progression.	[27]
E2	Viral regulatory gene	Represses E6/E7 expression; its disruption during viral integration leads to unchecked oncoprotein activity.	[28,29]
*TP53* (p53)	Host tumor suppressor	Governs DNA repair and apoptosis; degraded by E6, leading to genomic instability.	[27]
*RB1* (pRb)	Host tumor suppressor	Controls G1/S cell cycle checkpoint; inactivated by E7, releasing E2F and driving DNA synthesis.	[27]
*FAM19A4*	Host tumor suppressor	Frequently hypermethylated in high-grade lesions and cervical cancer; marker for disease progression.	[75,76,77,78]
*CADM1*	Host tumor suppressor	Hypermethylated in CIN3 and invasive cancers; associated with loss of cell adhesion and tumor progression.	[75,76,77,78]
*MAL*	Host tumor suppressor	Silenced via promoter hypermethylation; contributes to loss of epithelial differentiation.	[75,76,77,78]
miR124-2	Host microRNA	Epigenetically silenced in high-grade lesions; regulates gene networks controlling proliferation and apoptosis.	[75,76,77,78]
Genes involved in oxidative stress, DNA repair, and immune response	Host defense genes	Polymorphisms modulate susceptibility to persistent HPV infection and lesion progression.	[30]
Chromosomal regions with deletions/amplifications/translocations	Structural alterations	Reflect genomic instability associated with viral integration events.	[29]

## 5. HPV Methylation and Its Role in Cervical Carcinogenesis

Among the various epigenetic alterations induced by human papillomavirus (HPV), DNA methylation represents one of the most decisive and diagnostically relevant layers of regulation. Methylation of HPV DNA, particularly at CpG sites within the L1 and L2 capsid genes and the long control region (LCR), plays a central role in modulating viral transcription, replication, and integration [79].

The viral oncoproteins E6 and E7 can upregulate host DNA methyltransferases (DNMTs), promoting widespread methylation patterns that not only silence host tumor suppressor genes but also alter viral gene expression. For instance, methylation of regulatory sequences can repress the viral E2 gene, which normally inhibits E6 and E7, leading to sustained oncoprotein activity and enhanced cellular proliferation [80]. This interplay between viral methylation and oncoprotein-driven DNMT activity creates an epigenetic environment favorable to viral persistence, host genomic instability, and neoplastic progression.

In parallel, HPV infection induces extensive methylation changes within the host genome that directly contribute to the development of high-grade lesions and cervical cancer. Hypermethylation of tumor suppressor genes—including CADM1, MAL, FAM19A4, and miR-124-2—is a hallmark of high-grade cervical intraepithelial neoplasia (CIN) and invasive carcinoma, reflecting HPV-driven silencing of pathways involved in apoptosis, cell adhesion, and cell-cycle regulation [75,76]. Additional host genes, such as DPP6, RALYL, and GSX1, also display aberrant hypermethylation in HPV-positive high-grade lesions and invasive cancers, further disrupting regulatory networks governing cell-cycle checkpoints, cellular differentiation, and apoptotic control [80]. These methylation alterations accumulate progressively with lesion severity, functioning both as mechanistic drivers of carcinogenesis and as stable molecular signatures that reflect disease stage.

The degree of HPV DNA methylation correlates strongly with lesion severity, highlighting its value as both a mechanistic biomarker and a diagnostic tool. High methylation levels in the viral L1 and LCR regions are consistently associated with CIN2/3 and invasive cancer, distinguishing them from low-grade lesions or transient infections [23,79]. In particular, methylation of the L1 and L2 regions in high-risk HPV types such as HPV16, HPV18, HPV31, HPV33, and HPV45 shows a strong positive association with advanced lesions like CIN3 and adenocarcinoma in situ (AIS), underscoring its biological relevance in HPV-driven carcinogenesis. These methylation signatures are closely linked to viral integration events, which further compromise host genomic integrity and accelerate malignant transformation [81]. Importantly, host gene methylation patterns are highly stable and readily detectable in cervical samples, making them promising biomarkers for early detection, prognosis, and risk stratification [82].

Several host tumor suppressor genes exhibit consistent and robust methylation changes in high-grade cervical lesions and invasive cervical cancer. PAX1 shows significantly higher promoter methylation levels in high-grade squamous intraepithelial lesions (HSIL) and invasive cervical cancer (ICC) compared with normal cervical tissue (9–32% vs. 2–7%). Meta-analyses indicate that PAX1 methylation detects CIN2+ lesions with a pooled sensitivity of approximately 66% and specificity of 92%, potentially reducing unnecessary colposcopy referrals by up to 60% when applied as a triage tool, particularly in women with non-16/18 high-risk HPV infections. Most validation studies have been conducted in Asian populations, where PAX1 methylation has demonstrated higher specificity than HPV genotyping for identifying CIN3+ lesions, suggesting a potential role in reducing false-positive screening results [83,84,85].

Meta-analyses further show that CADM1 promoter methylation occurs significantly more frequently in cervical cancer tissues than in healthy controls, with a pooled odds ratio (OR) of 16.62, indicating a strong association with the malignant phenotype. Increased CADM1 methylation is also observed in precancerous lesions, including HSIL (OR 4.82) and LSIL (OR 2.30), supporting the notion that epigenetic silencing of this gene arises early during cervical carcinogenesis [83,85]. For MAL, pooled analyses reveal substantially higher methylation levels in cancerous tissues compared with normal controls (OR 31.06), consistent with a strong association with invasive disease. In contrast, MAL methylation does not differ significantly in LSIL (OR 1.32), while an association is observed in HSIL, albeit with considerable between-study heterogeneity (OR 5.23; I^2^ = 78%), highlighting variability in its performance across lesion grades [83,85]. POU4F3, a transcription factor located on chromosome 5q31–q33, also demonstrates progressive increases in methylation with lesion severity and widespread hypermethylation in both squamous cell carcinoma and adenocarcinoma. The multicenter TRACE study showed that POU4F3 methylation improves detection of CIN2+ lesions in hrHPV-positive women, achieving higher sensitivity than cytology (70.1% vs. 42.7%) while maintaining comparable specificity. Overall, reported sensitivities and specificities of up to 88% and 89%, respectively, indicate strong diagnostic potential, although further validation across diverse populations is warranted [83,84]. Other promising single-gene markers include SOX1 and CCNA1, which often outperform HPV DNA testing in specificity for HSIL+ detection. ZNF582, located on chromosome 19q13.43, exhibits a near-universal methylation in squamous cell carcinoma and substantial hypermethylation in adenocarcinoma (29–66%). High methylation correlates strongly with CIN3+ risk (OR = 15.52). When combined with HPV16/18 testing, ZNF582 methylation achieves excellent sensitivity (85.4%) and specificity (81.1%), effectively lowering colposcopy referrals [84].

## 6. DNA Methylation-Based Cervical Diagnostics

### 6.1. DNA Methylation as a Biomarker for CIN and Cervical Cancer

HPV methylation analysis is increasingly recognized as a powerful adjunct in screening, triage, and management of cervical intraepithelial lesions. Methylation assays can reliably differentiate transient infections from high-risk lesions requiring intervention, demonstrating high sensitivity and specificity [23,80]. When combined with host-gene methylation markers and HPV genotyping, these assays enhance the detection of CIN3 and invasive cancer while reducing unnecessary colposcopies. Lesions with high viral or host methylation are more likely to progress, supporting personalized management strategies: low-methylation lesions may be safely monitored, whereas high-methylation lesions warrant timely treatment [86]. Together, viral and host methylation signatures constitute a robust molecular toolkit, improving diagnostic precision and enabling more efficient, individualized cervical cancer prevention strategies. Both single-gene and multi-gene methylation markers demonstrate substantial potential to complement HPV testing and cytology, although further large-scale, multicenter validation is required to establish standardized clinical protocols.

Multi-gene methylation panels generally provide greater diagnostic accuracy and operational efficiency than single markers, particularly for triage and reduction of unnecessary colposcopy referrals. For example, combined methylation of CADM1/MAL correlates with cervical lesion severity and is positively associated with the duration of persistent HPV infection, making this panel useful for monitoring lesion dynamics over time. The FAM19A4/miR124-2 panel is independent of HPV genotype and detects nearly all cervical carcinomas, including rare histological subtypes and hrHPV-negative cases, with a reported positivity rate of 98.3%. When applied to women with ASC-US or LSIL cytology, this assay reduces colposcopy referrals by approximately 60% while maintaining high sensitivity for CIN3+ lesions (70.2%). Moreover, negative test results reliably predict lesion regression, providing a high negative predictive value for disease progression and correlating with a lower long-term risk of cervical cancer compared with negative cytology. The combined PAX1/SOX1 methylation panel has demonstrated strong performance in distinguishing LSIL from HSIL, achieving reported sensitivity and specificity of 100% and 95.7%, respectively, in Chinese cohorts. Its positive predictive value exceeds that of HPV testing alone, and integration with HPV genotyping further improves PPV to 81.8%. JAM3-based panels, particularly when combined with PAX1 or EPB41L3, also show enhanced diagnostic performance. For instance, the PAX1/JAM3 panel achieves an area under the curve (AUC) of 0.866 and reduces colposcopy referrals to 17.45%, while EPB41L3/JAM3 demonstrates 72.1% sensitivity and 91.5% specificity for CIN2+ detection. Additional multi-gene panels incorporating markers such as FMN2, EDNRB, ZNF671, TBXT, and MOS have shown the ability to detect HSIL and squamous cell carcinoma even in hrHPV-negative cases. Integrated approaches combining HPV16/18 genotyping with methylation-based triage strategies (e.g., PAX1/ZNF582) achieve balanced performance, with reported sensitivity and specificity of 78.9% and 73.6%, respectively, for CIN3+ detection, while minimizing unnecessary clinical interventions. Finally, the S5 classifier, which integrates methylation markers from the host gene EPB41L3 and late-region (L1/L2) viral genes from HPV16, HPV18, HPV31, and HPV33, provides a comprehensive molecular signature with higher sensitivity and comparable specificity relative to HPV16/18 partial genotyping for CIN2+ detection, thereby enhancing the accuracy of cervical cancer screening and triage [83,84,87]. Numerous approaches have been developed to identify and characterize DNA methylation patterns, such as Southern blot, methylation-specific PCR, and methylated DNA immunoprecipitation. A widely used preparatory method for many of these approaches is sodium bisulfite treatment, which chemically converts unmethylated cytosines to uracil, while leaving methylated ones unchanged. This conversion enables downstream assays to discriminate between methylated and unmethylated cytosines with high specificity. One of the most widely used commercial kits for bisulfite conversion of DNA is the “EZ DNA Methylation Kit” (Zymo Research, Irvine, CA, USA) [88,89,90,91,92], as well as EpiTect Bisulfite Kit (Qiagen) [93,94,95,96], CpGenome Turbo Bisulfite Modification Kit (Millipore, Burlington, MA, USA) [97,98,99,100], and Fast Bisulfite Conversion Kit (Abcam, Cambridge, UK) [98,101,102].

For CIN screening triage, the most widely recognized commercially available DNA methylation-based diagnostic assays are: GynTect^®^ (Oncgnostics), based on methylation-specific real-time PCR for six gene promoters (ASTN1, DLX1, ITGA4, RXFP3, SOX17, ZNF671). Similarly, QIAsure Methylation Test (QIAGEN) analyses bisulfite-converted DNA from cervical or vaginal samples and targets methylation of FAM19A4 and hsa-miR-124-2 promoters in HPV-positive women to identify “advanced transforming” CIN lesions (Table 2). Both kits are CE-marked (approved for in vitro diagnostics, IVD) for triage of HPV-positive women [103]. Importantly, methylation-based triage offers advantages: it is objective, can be done on the same sample collected for HPV screening (including self-sample specimens), and may reduce unnecessary colposcopies or overtreatment by better discriminating low-risk from high-risk lesions [104]. Additionally, the “S5 DNA-methylation classifier (S5^®^)”, a research-use methylation assay developed by Queen Mary University of London, analyzes DNA methylation of the host gene EPB41L3 and the late regions (L1 and/or L2) of high-risk HPV types 16, 18, 31, and 33. The assay has been evaluated in multiple studies as a molecular triage tool for detecting high-grade cervical lesions [105,106,107,108,109,110].

Beyond gene-specific methylation, global DNA methylation analysis provides a comprehensive view of epigenetic alterations associated with early cervical carcinogenesis. While gene-specific methylation assays target particular genes to detect high-grade lesions, global methylation reflects genome-wide changes, including hypomethylation of repetitive elements and overall genomic instability, which are hallmarks of neoplastic transformation. Assessing global DNA methylation can reveal epigenetic dysregulation affecting multiple pathways, offering insight into early disease processes that may not be captured by single-gene assays. ELISA-based kits for global methylation are particularly useful for rapid screening and longitudinal monitoring, as they can identify women at higher risk of CIN progression by detecting broad changes in methylation patterns [129]. Accordingly, multiple commercial kits are available to facilitate this analysis, such as “Global DNA Methylation ELISA” (Cell Biolabs, San Diego, CA, USA) [130,131,132,133], “Methylated DNA Quantification Kit” (Abcam) [134,135,136,137], “5-mC DNA ELISA Kit” (Zymo research) [138,139,140,141], “MethylFlash Methylated DNA5-mC Quantification Kit” (Epigentik, Farmingdale, NY, USA) [142,143,144,145,146].

Collectively, these methylation-based assays represent an important advancement in molecular diagnostics, enabling more precise identification of women at risk for cervical cancer and improving the overall effectiveness of screening programs.

### 6.2. Self-Sampling for Cervical Screening

The applicability of DNA methylation analysis is further enhanced by self-sampling strategies, which allow women to collect their own cervical or vaginal specimens. Self-sampling has emerged as an effective strategy to increase participation in cervical cancer screening, particularly among underscreened women. Studies have shown that women are twice as likely to participate when self-sampling is offered compared with standard clinician-based screening [147]. Self-collection can be performed at home or in a clinic, using a variety of devices such as swabs, cervical brushes, tampons, or cervico-vaginal lavages, and the specimens are then sent to a laboratory for analysis. Swabs and brushes are small, easy to manipulate, and processable like clinician-collected samples, while tampons and lavages require more extensive processing but are generally acceptable to women. hrHPV types show similar affinity for vaginal and cervical epithelium, making self-collected samples representative of cervical HPV status [148].

### 6.3. Clinical Performance

PCR-based hrHPV testing on self-collected specimens demonstrates high sensitivity and specificity for the detection of high-grade cervical lesions (CIN2+/CIN3+), comparable to clinician-collected samples [149,150,151]. While cytology cannot be reliably performed on self-collected samples, methylation marker analysis has shown good clinical performance for CIN3+ as a triage test for HPV-positive samples [152]. Overall, self-sampling reduces barriers related to shame, discomfort, and accessibility, decreases the number of healthcare visits, and effectively identifies women at increased risk, particularly among those who do not participate in routine screening programs [153]. 

### 6.4. Implementation and Acceptance

The implementation of self-sampling has been adopted in several countries and is widely accepted by women. Offering hrHPV self-sampling has been shown to increase screening coverage, particularly in rural and underscreened populations, and provides a practical strategy for reaching non-attending women, who often have a higher prevalence of high-risk HPV infections and an increased incidence of cervical cancer [147,153]. Self-sampling therefore represents not only a complementary approach to clinician-based screening but, in certain populations, a potentially preferable option for primary cervical cancer prevention. Table 3 summarizes the current landscape of commercially available self-sampling devices for cervical specimen collection. Among these, brush- and swab-based devices are the most widely used and clinically validated. The Evalyn^®^ Brush (Rovers Medical Devices) has been extensively studied and is implemented in several European screening programs, while the Digene Cervical Sampler (Qiagen) and the Cervex-Brush^®^ (Thermo Fisher Scientific) are commonly used for both clinician- and self-collected samples. Frequently used swab-based devices include HerSwab (Eve Medical) and FLOQSwabs^®^ (Copan), which are user-friendly and compatible with mail-in self-sampling protocols. Lavage-based devices, such as the Delphi Screener (Delphi Bioscience), are primarily employed in research settings, whereas other emerging devices—including Qvintip (Aprovix), Just For Me (Preventive Oncology International), V-Veil UP2™ (V-Veil-Up Pharma), HygeiaTouch Self Sampling (HygeiaTouch), and CERVICHECK™ (Pragmatech Healthcare Solutions)—are currently less widely adopted. Overall, brush- and swab-based devices dominate both clinical and research applications due to their ease of use, reliable cellular yield, and compatibility with molecular testing, while alternative designs remain largely limited to niche or investigational use.

## 7. Conclusions

Cervical cancer represents a paradigmatic model of virus-driven carcinogenesis in which viral, genetic, and epigenetic factors interact to influence susceptibility, disease initiation, and progression. Although persistent infection with high-risk HPV types is the primary causal event, host genetic predisposition—particularly polymorphisms in the HLA region and genes involved in immune regulation, DNA repair, and cellular homeostasis—modulates viral persistence and the likelihood of progression to neoplasia. Contemporary GWAS across diverse populations have identified both shared and population-specific susceptibility loci, reinforcing the multifactorial nature of cervical carcinogenesis and highlighting biological pathways beyond the classical HPV–host interaction.

At the molecular level, the HPV E6 and E7 oncoproteins disrupt cellular homeostasis by inactivating key tumor suppressor pathways, promoting genomic instability, and impairing DNA damage responses. In parallel, these oncoproteins drive extensive epigenetic reprogramming through alterations in DNA methylation, histone modifications, and non-coding RNA networks. Together, these changes establish permissive transcriptional programs that facilitate the progression from low-grade lesions to CIN2/3 and invasive disease.

Among epigenetic mechanisms, DNA methylation has emerged as one of the most consistently associated molecular features of transforming HPV infections. Hypermethylation of host genes such as *CADM1*, *MAL*, *FAM19A4*, miR-124-2, *PAX1*, *SOX1*, and *ZNF582*, as well as methylation of viral genomic regions, correlates with lesion severity and reflects underlying biological processes of malignant transformation. These observations have stimulated the development of methylation-based assays for use as adjunctive triage tools following a positive HPV test. However, while multiple assays demonstrate promising diagnostic performance in research and selected clinical settings, variability in sensitivity, specificity, and reproducibility across studies currently limits the ability to recommend any single biomarker or assay for routine clinical implementation.

Advances in self-sampling have clearly strengthened molecular screening strategies. Self-collected cervical or vaginal samples are well suited for hrHPV testing and compatible with downstream molecular analyses, offering performance comparable to clinician-collected samples while addressing barriers related to access, acceptability, and screening participation. At present, HPV testing, particularly when combined with self-sampling, remains the only fully validated molecular approach for population-level cervical cancer screening.

Overall, the integration of genetic insights, epigenetic profiling, and modern molecular technologies has substantially advanced our understanding of HPV-driven cervical carcinogenesis. Nevertheless, the translation of genetic and epigenetic biomarkers into clinical practice remains constrained by unresolved challenges. These include heterogeneity in biomarker performance across populations, limited reproducibility between studies, and a lack of formal comparative and cost-effectiveness analyses, particularly in low- and middle-income countries where disease burden is highest. Large prospective studies, standardized assay protocols, and rigorous health-economic evaluations will be essential to determine whether methylation-based or other molecular biomarkers can move beyond the research setting and meaningfully complement established HPV-based screening strategies in global cervical cancer prevention efforts.

## Figures and Tables

**Table 2:** Overview of commercially available DNA Methylation Detection-Based kits for CIN.

Manufacturer Company	Kit/Device Name	Country of Origin	Target Sites	References
Qiagen	QIAsure Methylation Test	Hilden, Germany	FAM19A4 and hsa-mir124-2	[76,107,111,112,113,114]
Oncgnostics GmbH	GynTect^®^ assay	Jena, Germany	ASTN1, DLX1, ITGA4, RXFP3, SOX17, ZNF671	[107,112,113,115,116,117,118]
Fujirebio	PreCursor-M+	Tokyo, Japan	FAM19A4 and miR124-2	[119,120,121,122]
LifeGene BioMarks	CervicalMethDx	Baltimore, MD, USA/Toa Baja, PR, USA	ZNF516, FKBP6, and INTS1	[123]
Hybribio	Methylation Real-time PCR Kit	Hong Kong, China	SOX1 and PAX1	[124,125]
Beijing SinoMDgene Technology Co.	Methylated Human *EPB41L3* and *JAM3* Gene Detection Kit	Beijing, China	EPB41L3 and JAM3	[126,127]
OriginPoly Bio-Tec	CISCER or Human PAX1 and JAM3 Gene Methylation Detection kit	Beijing, China	PAX1 and JAM3	[128]
Origin Biotechnology	Human *PAX1* and *JAM3* Gene Methylation Detection Kit	Xiamen, China	PAX1 and JAM3	[129]
Oncgnostics GmbH	ScreenYu Gyn^®^	Jena, Germany	one human gene region	-
careLYFE	careREVEAL	Suzhou, China	host gene EPB41L3 and hrHPV genes	-
Shanghai GeneoDx Biotech Co	DNA Methylation Detection Kit	Shanghai, China	ASTN1/DLX1/ITGA4/RXFP3/SOX17/ZNF671	-
YanengBio	DNA Methylation Detection Kit	Shenzhen, China	PAX1, SOX1 and HAS1	-

**Table 3:** Commercially Available Self-Sampling Devices for Cervical Specimen Collection.

Manufacturer Company	Kit/Device Name	Country of Origin	Self-Sample Device	References
Rovers Medical Devices	Evalyn Brush	Oss, The Netherlands	Brush	[88,150,154,155,156,157,158,159,160,161,162,163,164,165,166,167]
Qiagen	Digene cervical sampler	Hilden, Germany	Brush	[168,169,170,171,172,173,174,175,176,177]
Rovers Medical Devices	Viba-brush	Oss, The Netherlands	Brush	[178,179,180,181,182,183,184,185,186,187]
Delphi Bioscience BV (formerly Pantarhei Devices)	Delphi Screener	Scherpenzeel, The Netherlands	Lavage	[89,90,151,155,161,188,189,190,191]
Aprovix AB	Qvintip self-sampling kit	Solna, Sweden	Wand/Swab	[153,191,192,193,194,195,196,197]
Thermo Fisher Scientific Inc.	Cervex-Brush Cervical Cell Sampler	Waltham, MA, USA	Brush	[198,199,200,201,202,203,204,205]
Eve Medical	HerSwab	Toronto, ON, Canada	Swab	[165,191,193,195,206,207,208]
Medical Packaging Corporation	Dacron polyester swab	Camarillo, CA, USA	Swab	[175,209,210,211,212,213]
Copan	FLOQSwabs	Brescia, Italy	Swab	[156,159,163,214,215,216]
Preventive Oncology International	Just For Me	Cleveland Heights, OH, USA	Brush	[217,218,219,220,221]
Teal Health, Inc.	Teal Wand	San Francisco, CA, USA	Wand	[222,223,224,225,226]
CooperSurgical	Cytobrush	Trumbull, CT, USA	Brush	[200,201,227,228]
V-Veil-Up Pharma	Vaginal Veil Collector V-Veil UP2™ device	Pitesti, Romania	Veil	[229,230,231]
Hygeia Touch Inc.	HygeiaTouch Self Sampling	Hazle Township, PA, USA	Stick	[232,233,234]
Pragmatech Healthcare Solutions	CERVICHECK™ Self-Sampling Kit	Vadodara, India	Brush	[235]

## Data Availability

No new data were created or analyzed in this study. Data sharing is not applicable to this article.
